# AN EMBEDDED MIXED METHODS EVALUATION OF THE RESIDENTIAL CARE TRANSITION MODULE

**DOI:** 10.1093/geroni/igae098.1258

**Published:** 2024-12-31

**Authors:** Robyn Birkeland, Joseph Gaugler, Elizabeth Albers, Colleen Peterson, Katie Louwagie, Zachary Baker, Mary Mittelman

**Affiliations:** University of Minnesota, Minneapolis, Minnesota, United States; University of Minnesota, Minneapolis, Minnesota, United States; University of Minnesota, Minneapolis, Minnesota, United States; University of Michigan Transportation Research Institute, Ann Arbor, Michigan, United States; University of Minnesota, Minneapolis, Minnesota, United States; Arizona State University, Phoenix, Arizona, United States; New York University, New York, New York, United States

## Abstract

Mixed methods designs refer to those research strategies that integrate or “mix” qualitative and quantitative research questions, sampling and data collection approaches, and analyses, so that one component builds upon and expands the inferences of the other. The Residential Care Transition Module (RCTM) evaluation relied on an “embedded” mixed methods design. In the RCTM embedded design, qualitative data collection was integrated during and following intervention delivery to ascertain components or contextual factors that may have influenced participant benefit. We relied on the robust qualitative data available from quarterly review checklists as well as purposive, post-intervention semi-structured interviews administered to RCTM treatment participants to identify and empirically test three mediators and moderators in a series of post-hoc, longitudinal quantitative analyses: unemployment, time in residential care, and bereavement status. In this presentation, we will highlight our findings for whom and under what circumstances a telehealth dementia care support intervention such as the RCTM may exert positive benefits.

